# Nitrate alleviates ammonium toxicity in wheat (*Triticum aestivum* L.) by regulating tricarboxylic acid cycle and reducing rhizospheric acidification and oxidative damage

**DOI:** 10.1080/15592324.2021.1991687

**Published:** 2021-11-09

**Authors:** Wanying Du, Yunxiu Zhang, Jisheng Si, Yan Zhang, Shoujin Fan, Haiyong Xia, Lingan Kong

**Affiliations:** a College of Life Science, Shandong Normal University, Jinan, China; b Crop Research Institute, Shandong Academy of Agricultural Sciences, Jinan, China

**Keywords:** Ammonium toxicity, H^+^ efflux, nitrate, root, tricarboxylic acid cycle, wheat

## Abstract

Ammonium (NH_4_^+^) is one of the most important nutrients required by plants. However, a high concentration of NH_4_^+^ as the sole nitrogen source suppresses plant growth. Although nitrate (NO_3_^−^) can alleviate NH_4_^+^ toxicity, the mechanisms underlying this ability have not been fully elucidated. In this study, wheat plants were cultivated in hydroponic solution with 7.5 mM NO_3_^−^ (control), 7.5 mM NH_4_^+^ (sole ammonium, SA) or 7.5 mM NH_4_^+^ plus 1.0 mM NO_3_^−^ (ammonium and nitrate, AN). The results showed that compared with the control, the SA treatment significantly decreased root growth, protein content and the concentrations of most intermediates and the activity of enzymes from the tricarboxylic acid (TCA) cycle. Moreover, increased the activity of plasma membrane H^+^-ATPase and the rate of H^+^ efflux along roots, caused solution acidification, and increased the activity of mitochondrial respiratory chain complexes I–IV and the contents of protein-bound carbonyls and malondialdehyde in roots. SA treatment induced ultrastructure disruption and reduced the viability of root cells. Compared with the SA treatment, the AN treatment increased root growth, protein content, the concentrations of most intermediates and the activity of enzymes from the TCA cycle. Furthermore, AN treatment decreased the rate of H^+^ efflux, retarded medium acidification, decreased protein carbonylation and lipid peroxidation in roots and relieved ultrastructure disruption and increased the viability of root cells. Taken together, these results indicate that NO_3_^–^-dependent alleviation of NH_4_^+^ toxicity in wheat seedlings is closely associated with physiological processes that mediate TCA cycle, relieve rhizospheric acidification and decrease the production of ROS and oxidative damage.

## Introduction

Nitrogen (N) is an important nutrient for plant growth. Ammonium (NH_4_^+^) and nitrate (NO_3_^−^) are the two forms of inorganic N that can be taken up by plants. Compared with NO_3_^–^-N, NH_4_^+^-N is a preferred N source for many crops because NH_4_^+^ can be directly converted into amino acids and requires less energy for both transport and assimilation.^1,2^ However, the application of excessive NH_4_^+^ decreases the activities of antioxidant enzymes in *Eustoma grandiflorum, Myriophyllum aquaticum* and *Panax notoginseng*^3–5^ and induces rhizosphere acidification in rice (*Oryza sativa* L.) and Arabidopsis (*Arabidopsis thaliana*) seedlings.^6,7^ High doses of NH_4_^+^ may restrict carbon availability and inhibit the rate of net photosynthesis.^8^ High NH_4_^+^ concentrations in plants increase electrolyte leakage and cause nutritional imbalances of cations in cells, such as decreased concentrations of K^+^, Ca^2+^ and Mg^2+^.^9–11^ In particular, root growth was significantly reduced in Arabidopsis, sugar beet (*Beta vulgaris* L.), rice and spinach (*Spinacia oleracea* L.) when plants were exposed to higher concentrations of NH_4_^+^.^11–13^ Nevertheless, to date, little is known about the detailed mechanisms of NH_4_^+^ toxicity.^13^

An increasing number of studies have been conducted to investigate the mechanisms underlying which exogenous substances alleviate NH_4_^+^ stress. Silicon alleviated NH_4_^+^ toxicity in cauliflower (*Brassica oleracea*) through an increase in the physical integrity of the membranes^14^ and promoted sugar beet (*Beta vulgaris* L.) growth.^12^ Increasing the carbon supply allowed the adjusted regulation of root defense mechanisms by enhancing amino acid synthesis in tomato (*Solanum lycopersicum* L.) when dealing with NH_4_^+^ toxicity.^15^ Supplementation with *α*-ketoglutarate (KGA), a key carbon source for N assimilation, alleviated toxicity symptoms in rice.^16^ Small amounts of NO_3_^−^ can effectively alleviate NH_4_^+^ toxicity in many plants.^9^ For example, low concentration of NO_3_^−^ can improve survival and growth of cucumber (*Cucumis sativus* L.) seedlings under NH_4_^+^ toxicity by acting as a ‘signal molecule’ to promote cytokinin synthesis in roots and cytokinin translocation from roots to shoots.^17^ It was found that nitrate alleviated the toxic effect of NH_4_^+^ on root growth of *Panax notoginseng* by up-regulation of *ACLA-3* (encoding ATP-citrate lyase A-3) and several key metabolites in the tricarboxylic acid (TCA) cycle.^18^ SLAH3, a slow-type NO_3_^−^ efflux channel, mediates the NO_3_^−^ efflux to buffer the low cellular pH caused by NH_4_^+^ uptake.^6^ SnRK1.1 can regulate SLAH3 and thereby mediate NO_3_^−^ efflux, leading to alleviation of high-NH_4_^+^/low-pH stress.^13^ Several studies suggest that NO_3_^−^ alleviate NH_4_^+^ toxicity, likely as a signal. However, how NO_3_^−^ signaling alleviates NH_4_^+^ toxicity is far from clear.^13,19^

The root is an important organ of NH_4_^+^ uptake and assimilation, and its development is strongly affected by NH_4_^+^.^20^ Thus, analysis of the root responses is essential to understand NH_4_^+^ toxicity and underlying mechanisms of NO_3_^–^dependent mitigation of the toxicity. In a recent work, we found that shoot growth of wheat seedlings was not affected under sole NH_4_^+^ nutrition.^21^ Therefore, the objectives of the present study were to investigate and elucidate the mechanisms driving NH_4_^+^ toxicity and how NO_3_^−^ mitigates NH_4_^+^ toxicity in roots of wheat. Our results suggest that nitrate-dependent alleviation of NH_4_^+^ toxicity in wheat were closely associated with activation of TCA cycle, supplement of carbon skeletons, mitigation of the rhizospheric acidification and oxidative damage on lipids and proteins. Based on these results, we discussed the possible physiological mechanisms underlying nitrate-dependent alleviation of NH_4_^+^ toxicity. We anticipate that the findings of this study will be helpful to explore new methods for applying N fertilizers to alleviate NH_4_^+^ stress in the field and to improve N use efficiency.

## Materials and methods

### Plant growth and culture conditions

Wheat (Jimai22) seeds were sterilized with 70% (v/v) ethanol for 45 s and then rinsed three times with distilled water and germinated at 25°C for 48 h in petri dishes that were covered with wet sterilized gauze. Fifteen three-day-old seedlings were transplanted into a growth chamber at 25/21°C under a 14 h day/10 h night photoperiod; the light intensity was 6000 µmol m^−2^ s^−1^, and the relative humidity was maintained at 70 ± 5% during day and night. The 15 seedlings were fixed in plastic pots (10 cm × 8 cm × 5 cm in length, width, and height, respectively) and cultured in distilled water. The distilled water was renewed every two days. The plastic pots were randomly placed, and their positions were changed frequently. In our previous study, we found that 8–15 d old wheat seedlings grew best in half-strength Hoagland’s nutrient solution, and addition of 1 mM NO_3_^−^ was most effective in alleviating 7.5 mM NH_4_^+^ stress.^21^ After reserve depletion, eight-day-old seedlings were cultured in half-strength Hoagland’s nutrient solution.^22^ After 48 h, the wheat seedlings were treated with half-strength Hoagland’s nutrient solution containing 7.5 mM NO_3_^−^ (control; applied as KNO_3_ and Ca(NO_3_)_2_ at 2.5 mM), 7.5 mM NH_4_^+^ (SA; applied as 7.5 mM NH_4_Cl) or 7.5 mM NH_4_^+^ plus 1 mM NO_3_^−^ (AN; applied as 6.5 mM NH_4_Cl and 1 mM NH_4_NO_3_). The Ca^2+^ and K^+^ concentrations were balanced by the addition of CaCl_2_ and K_2_SO_4_.

### Measurement of root Fresh Weight (FW)

Roots of wheat seedlings were collected from each treatment and weighed immediately at 0, 24, 48, 72 and 96 h after treatment. The net increase in root FW from 0 to 24, 48, 72 or 96 h after treatment was calculated. The measurements were repeated on three independent biological experiments, each in triplicate with 15 plants.

### Determination of NH_4_^+^ and protein content

Wheat roots (approximately 50.0 mg) were harvested and desorbed for 5 min in 10 mM CaSO_4_ to remove extracellular NH_4_^+^. The samples were ground in liquid N, and then 6 mL of 10 mM formic acid was added to extract NH_4_^+^. The homogenate was centrifuged at 10,000 g at 4°C for 10 min. The supernatant was transferred into a tube and centrifuged at 20,000 g and 4°C for 10 min. The *o*-phthalaldehyde method was used to determine root NH_4_^+^ content.^23^ Briefly, 100 mL of *o*-phthalaldehyde (OPA) reagent was prepared by combining 200 mM potassium phosphate buffer (KPB) (pH 7.2) and 3.75 mM OPA and the pH was adjusted to 7.0 with 1 M NaOH. Prior to the usage, the solution was added 2 mM 2-mercaptoethanol and filtered through filter paper. The 10 mL supernatant of extract was mixed with 2 mL of OPA reagent, then color was allowed to develop in the dark for 20 min at room temperature, and sample absorbance was measured at 410 nm using a spectrophotometer (Varian Cary 100, Australia).

Soluble protein content of root was determined using the method of Bradford.^24^ Roots (1.0 g) were homogenized with 50 mM KPB (pH 7.2) and the homogenate was centrifuged at 30,000 g at 4°C for 10 min then the supernatant was collected. The supernatant (1 mL) was mixed with 5 mL Coomassie Brilliant Blue G-250 regent (containing 0.01% (w/v) Coomassie Brilliant Blue G-250, 9.0% (v/v) ethanol, and 8.5% (w/v) H_3_PO_4_). Absorbance was measured at 595 nm using a spectrophotometer (Varian Cary 100, Australia) and soluble protein content was calculated from a standard curve obtained with bovine serum albumin (BSA).

### Determination of enzyme activities and intermediate concentrations of the TCA cycle

Fresh roots (approximately 1.0 g) were ground and homogenized in 1 mL extraction on ice. The homogenates were then placed into microcentrifuge tubes, and the supernatants (enzyme extracts) were obtained by centrifuging the homogenate at 8,000 g at 4°C for 15 min. The supernatants were used to determinate the activities of aconitase, citratesynthase (CS), a-ketoglutarate dehydrogenase (a-KGDH), succinic dehydrogenase (SDH), malate dehydrogenase (MDH), pyruvate carboxylase (PC) and pyruvate dehydrogenase (PDH) using commercial chemical assay kits (Comin, Suzhou Comin Biotechnology Co. Ltd., Suzhou, China), according to the manufacturer’s instructions. The absorbance was measured using a microplate reader (Rayto RT-6100, Rayto Corporation, Shenzhen, China), and the activity of the enzymes was calculated based on the fresh weight using the corresponding calculation formulas.

For determination of TCA cycle intermediates, fresh roots (approximately 0.1 g) were ground and homogenized in 1 mL extraction on ice. The homogenates were transferred tomicrocentrifuge tubes, centrifuged at 12,000 g at 4°C for 10 min, and then the supernatant was collected. The concentrations of aconitate, acetylcoenzymeA (acetyl-CoA), citric acid (CA), fumaric acid, malic acid, oxaloacetic acid (OAA) and succinic acid were determined using commercial chemical assay kits (Suzhou Comin Biotechnology Co., Ltd., Suzhou, China). The optic density value of the solution was measured with a spectrophotometer (VarianCary100, Australia). The concentrations of these intermediates were calculated according to the manufacturer’s directions.

### Assay of Plasma Membrane (PM) H^+^-ATPase activity and H^+^ flux

Wheat roots (approximately 1.0 g) were ground in a mortar with liquid N and a pestle. Then, the samples were homogenized in phosphate buffer (pH 7.4). The homogenate was centrifuged at 10,000 g for 10 min at 4°C to collect the supernatant, which was centrifuged again at 87,000 g for 35 min. PMs were obtained from the microsomal fraction by partitioning in aqueous dextran T-500 and polyethylene glycol in an aqueous polymer two-phase system. The activity of the PM H^+^-ATPase was assayed according to the method of Zhang et al.^25^ After the addition of 1–2 mg membrane resuspension that contained protein into the 1 mL reagent (contained 30 mM BTP/MES, 5 mM MgSO_4_, 50 mM KCl, 0.02% (w/v) Brij 58 (a polyoxyethylene acyl ether), and 5 mM disodium-ATP) to start the reaction, the mixture was incubated for 30 min at 30°C. To terminate the reaction, 1 mL of stop reagent [5% (w/v) sodium dodecyl sulfate, 0.7% (w/v) (NH_4_)_2_MoO_4_ and 2% (v/v) concentrated H_2_SO_4_] was added, and followed by addition of 50 μL of 10% (w/v) ascorbic acid. To examine the purity of the collected samples, 1.45 mL of a mixture of 2% (w/v) sodium citrate, 2% (w/v) glacial acetic acid, and 2% (w/v) sodium arsenite was used to suppress the detection of phosphate liberated from H^+^-ATPase activity and ATP hydrolysis under acidic conditions.^26^ The color development was stable for 30 min at room temperature. The absorbance was measured at 820 nm by a microplate reader (Rayto RT-6100, Rayto Corporation, Shenzhen, China), and the PM H^+^-ATPase activity was calculated according to a standard curve.

Net H^+^ flux was measured using noninvasive micro-test technology (NMT) (Xuyue (Beijing) Sci. & Tech. Co. Ltd.). The samples were measured in a solution (containing 0.1 mM KCl, 0.1 mM CaCl_2_, 0.1 mM MgCl_2_, 0.5 mM NaCl, 0.3 mM MES, and 0.2 mM Na_2_SO_4_) at pH 6.0. Fick’s first law of diffusion (*J = −D × ΔC/ΔX*) was used to calculate the H^+^ flux, where J is the free H^+^ flux (fmol cm^−2^ s^−1^), D is the molecular diffusion coefficient (7 × 10^−6^ cm^2^ s^−1^), ΔC is the H^+^ concentration gradient, and ΔX is the excursion distance (30 μm). The net flux of H^+^ at the meristem zone (between 0 and 2,400 µm from the root tip) was measured. Each seedling was measured once. The final flux values were the means of more than six individual plants from each treatment. H^+^ flux was recorded at a rate of one reading per 6 s. The net flux of H^+^ data was processed using Magefux (Younger USA Corp., https://youngerusa.com/mageflux) based on Fick’s law of diffusion.

### Measurement of medium pH

The medium pH was measured using a pH meter (PHS-3E, Shanghai, China) at 0, 6, 12, 24 and 48 h after continuous treatment without renewal of the medium. The measurement was repeated six times for each treatment.

### Assay of mitochondrial respiratory complex activity

The root cooled to 4°C and mitochondrial isolation was conducted at 4°C. All procedures for mitochondrial isolation were according to Eubel et al.^27^ Hypotonically isolated mitochondria from root cells were lysed on ice for 1 h in 2% (w/v) dodecyl maltoside and 0.4 M aminocaproic acid. Upon centrifugation at 30,000 g for 20 min at 4°C, the supernatant was used for the activity assay.

The activity of NADH dehydrogenase (complex I) was measured by the method of Verner et al.^28^ The supernatant (5 μL) and 20 mM NADH (5 μL) were mixed with 1 mL of NDH buffer (50 mM KPi, pH 7.6; 1 mM EDTA, pH 8.5; 0.2 mM KCN). The reaction was started by the addition of 2 mM coenzyme Q_2_ and followed at 340 nm for 3 min. The activity of complex I activity was expressed as nmol of NADH oxidized per min per g FW.

The activity of succinate dehydrogenase (complex II) was measured by the method of Sudheesh et al.^29^ The mitochondrial lysate (approximately 4.0 mg) was mixed with 3 mg/mL BSA, 2 mM EDTA, 2 mM KCN, 1 μM antimycin A, 1 μM rotenone, 20 mM sodium succinate, 65 μM decyl ubiquinone and 50 mM KPB (pH 7.2) at 30°C for 10 min. The reaction was initiated with the addition of 60 μM 2,6-dichloroindophenol and monitored at 600 nm for 5 min. The activity of complex II activity was expressed as nmol of DCIP consumed per min per g FW.

The activity of cytochrome-*c* reductase (complex III) was measured as described by Singh et al.,^30^ with minor modifications. Briefly, the mitochondrial lysate was mixed with 50 mM KPB (pH 7.6), 0.5 mM EDTA, 2 M KCN, 50 μM cytochrome *c*, 20 mM succinate, 1% BSA, and 2 μg/mL rotenone. The reaction was monitored at 550 nm for 1 min. The activity of complex III activity was expressed as the rate of nmol cytochrome *c* reduction per min per g FW.

The activity of cytochrome-*c* oxidase (complex IV) was measured as described by Hachiya et al.,^31^ with minor modifications. The wheat roots (approximately 0.1 g) were ground in liquid N and mixed with 100 mM KPB (pH 7.5) containing 250 mM sorbitol and 0.2 mM Na_2_EDTA. The homogenates were centrifuged at 3,000 g for 2 min, the supernatant was centrifuged at 15,000 g for 10 min, and the pellets were resuspended in 50 mM KPB buffer. Reduced cytochrome *c* was added to the buffer, and complex IV activity was measured as the oxidation rate of the reduced cytochrome *c* by the change in absorbance at 550 nm. The activity of complex IV activity was expressed as the oxidation rate of the nmol cytochrome *c* reduced per min per g FW.

### Determination of protein-bound carbonyls and malondialdehyde (MDA) content

Wheat roots (approximately 1.0 g) were ground in liquid N using a mortar and pestle. Nucleic acids from the root homogenates were removed by treatment with 1% (w/v) streptomycin sulfate. The sample homogenates (1 mL after treatment) were transferred into a tube and then blended with 4 mL of 10 mM 2,4-dinitrophenylhydrazine. The mixture was left at room temperature for 30 min, and 5 mL of 20% trichloroacetic acid was added to the tube. The tube was incubated in an ice bath for 10 min, followed by centrifugation at 10,000 g for 20 min. The protein pellet was removed, rinsed three times with an ethanol/ethyl acetate mixture (1/1, v/v) and dissolved in 6.0 M guanidine hydrochloride (pH 2.3). The absorbance was measured at 370 nm using a spectrophotometer (Varian Cary 100, Australia), and the carbonyl content was calculated using a molar absorption coefficient of 22,000 M^−1^ cm^−1^.

The MDA content was measured using the method of Bouabid et al.^32^ The roots (approximately 0.1 g) were ground with 10 mL 0.1% (w/v) trichloroethane. The extract was centrifuged at 12,000 g at 4°C for 10 min. The supernatant (1 mL) was collected and mixed with 3 mL 0.6% thiobarbituric acid and 7 mL of phosphoric acid 1%. The mixture was then heated in a water bath at 90°C for 30 min and allowed to cool, after which 20 mL of *n*-butanol was added. The reaction mixture was centrifuged at 3,000 g for 10 min, and the absorbance was read by a spectrophotometer (Varian Cary 100, Australia) at 539 nm.

### Transmission electron microscopy

After treatment for 2 days, root apex samples were cut into 1 mm pieces, rapidly fixed in 2.5% glutaraldehyde overnight at 4°C and then further fixed in 1% osmium tetroxide for 2 h. The samples were washed in distilled water three times and dehydrated in increasing ethanol concentrations and acetone. Then, the samples were infiltrated with Spurr’s resin. Samples were cut into 70 nm thick sections using an ultramicrotome (Leica EM UC6, Leica, Germany) and collected on Cu-based grids. The grids were stained with 4% uranyl acetate for 15 min and 3% lead citrate for 5 min. Sections were observed under a JEOL JEM-2100EX transmission electron microscope (Jeol, Tokyo, Japan) operated at 80 kV.

### Assessment of cell viability

Cell viability at the root apex was assessed using staining with propidium iodide (PI) (Sigma-Aldrich). The root tips were stained in 10 μM PI for 1 min in the dark at room temperature and then rinsed twice in distilled water. Stained root sections were mounted onto microscope slides in 10% (v/v) glycerol and were observed using a confocal microscope (Leica TCS SP8, Leica, Germany) with a Texas Red filter (excitation wavelength: 480 nm; emission wavelength: 610 nm).

## Statistical analysis

Data Processing System (DPS) statistical software (DPS 10.05, Hangzhou, Zhejiang, China) was used for statistical analyses. Tukey’s tests at *P* < .05 were used to evaluate the significance of the results.

## Results

### Effects of different N sources on root growth

**Figure 1.**
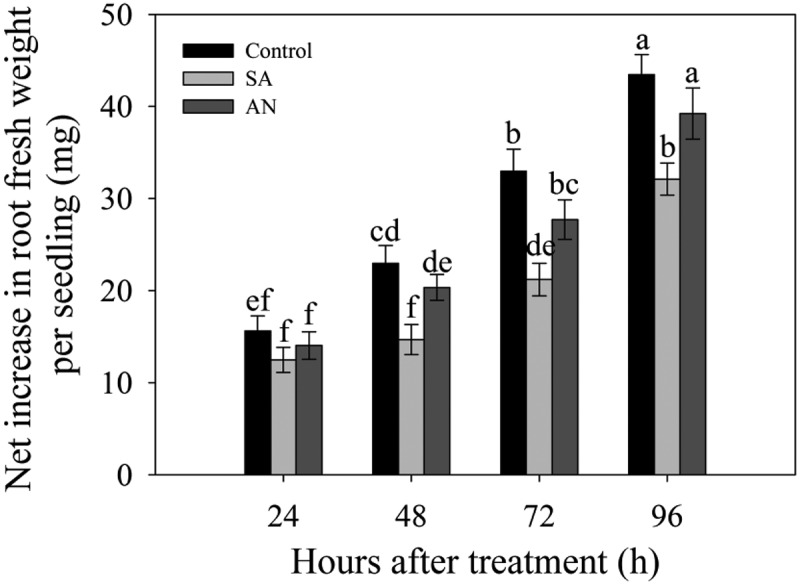
Effects of different N treatments on the root growth of wheat seedlings. The data show the means of the net increases in root fresh weight at 24, 48, 72 and 96 h. The results represent the mean ± SD of three independent experiments. Different lowercase letters above the columns indicate significant differences at *P* < .05. Control: wheat seedlings grown in 7.5 mM NO_3_^−^; SA: wheat seedlings grown in 7.5 mM NH_4_^+^; AN: wheat seedlings grown in 7.5 mM NH_4_^+^ + 1.0 mM NO_3_^−^.

At 24 h, no significant difference in the net increase in the fresh weight of the roots was observed between the control, SA and AN treatments. At 48, 72 and 96 h, the SA and AN treatments decreased the net increase in root fresh weight compared with the control. Meanwhile, the net increases under the AN treatment were higher than those under the SA treatment after 24 h (Figure 1).

### The content of NH_4_^+^ and protein in roots

**Figure 2.**
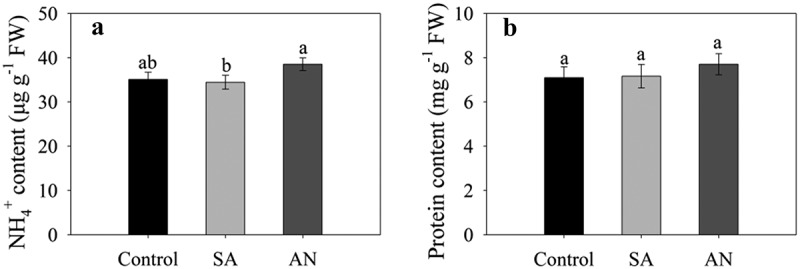
Effects of different N treatments on the contents of NH_4_^+^ (a) and protein (b) in wheat seedling roots. The results represent the mean ± SD of three independent experiments. Different lowercase letters above the columns indicate significant differences at *P* < .05. Control: wheat seedlings grown in 7.5 mM NO_3_^−^; SA: wheat seedlings grown in 7.5 mM NH_4_^+^; AN: wheat seedlings grown in 7.5 mM NH_4_^+^ + 1.0 mM NO_3_^−^.

No significant difference in the root NH_4_^+^ content in wheat seedlings was observed between the SA, AN and control conditions, while the AN treatment increased the root NH_4_^+^ content by 11.79% compared to the SA treatment (Figure 2a).

At 48 h, no significant difference in protein content of root was observed between SA and control. Moreover, AN treatment to a some extent increased the root protein content compared with the control and SA treatment (Figure 2b).

### The enzyme activity and concentration of intermediates of the TCA cycle

In the SA treatment, the activities of root CS, aconitase, *α*-KGDH, MDH and PDH were significantly decreased, while the activities of SDH and PC were increased in wheat seedlings compared with those in the control. In the AN treatment, the activities of root CS, aconitase, *α*-KGDH, MDH and PDH were greatly enhanced, while the activity of SDH was decreased compared with that in the SA treatment. The activities of CS, aconitase, *α*-KGDH, MDH, PDH and SDH in roots in the AN treatment were similar to those in the control.

**Figure 3.**
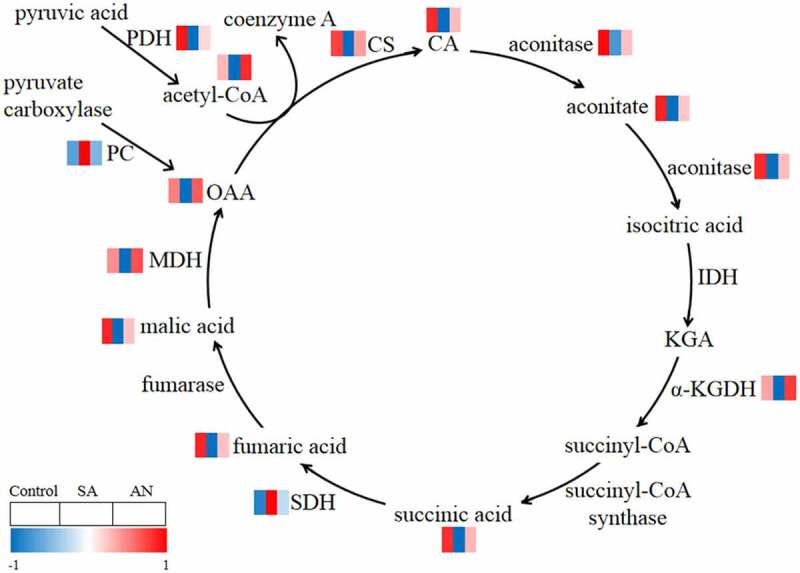
Reactions in the root TCA cycle of wheat seedlings treated with different N sources for 48 h. The levels of enzymes and the intermediates are shown by the color gradient, from low (blue) to high (red). The squares ordered from left to right indicate the treatments: 7.5 mM NO_3_^−^ (control), 7.5 mM NH_4_^+^ (SA), and 7.5 mM NH_4_^+^ + 1.0 mM NO_3_^−^ (AN). Acetyl-CoA, acetyl coenzyme a; CA, citric acid; CS, citrate synthase; IDH, isocitrate dehydrogenase; KGA, α-ketoglutarate; α-KGDH, α-ketoglutarate dehydrogenase; MDH, malate dehydrogenase; OAA, oxaloacetic acid; PC, pyruvate carboxylase; PDH, pyruvate dehydrogenase; SDH, succinic dehydrogenase.

The intermediates of the TCA cycle, including OAA, CA, aconitate, succinic acid, fumaric acid and acetyl-CoA, showed lower concentrations under the SA treatment than under the control conditions. After AN treatment, these intermediate concentrations were significantly higher than those in the SA treatment, while no significant differences were observed between the AN treatment and the control (Figure 3; Table S1).

**Table 1.** Activities of enzymes and concentrations of intermediates of three treatments

	control	SA	AN
citrate synthase	16.08±0.46^a^	5.40±0.46^c^	14.04±0.07^b^
citric acid	1088.39±18.40^a^	626.29±18.47^c^	953.69±17.34^b^
malate dehydrogenase	4120.54±98.01^a^	3657.82±106.69^b^	4239.72±96.65^a^
oxaloacetic acid	416.55±1.59^a^	392.39±1.71^b^	418.69±2.05^a^
fumaric acid	211.66±2.99^a^	106.70±2.95^c^	178.52±2.19^b^
α-ketoglutarate dehydrogenase	18.35±0.99^a^	12.40±0.32^b^	19.99±0.82^a^
succinic acid	576.72±5.34^a^	344.58±4.43^c^	509.43±12.98^b^
aconitase	27.13±0.73^a^	16.71±0.38^c^	24.03±0.35^b^
aconitate	1023.65±36.11^a^	738.85±14.45^c^	929.50±12.10^b^
succino dehydrogenase	2.28±0.01^c^	3.91±0.18^a^	2.78±0.17^b^
acetyl coenzyme A	20.78±0.92^a^	16.58±0.79^b^	22.44±1.27^a^
pyruvate dehydrogenase	3.82±0.15^a^	3.08±0.16^b^	3.54±0.18^a^
Pyruvate carboxylase	78.47±1.77^b^	90.95±3.06^a^	79.48±2.85^b^
malic acid	388.83±7.89^a^	118.41±1.08^c^	269.62±3.10^b^

Effects of different N treatments on activities of enzymes and concentrations of the contents of intermediates in wheat seedling roots. The results represent the mean ± SD of three independent experiments. Different lowercase letters in each row indicate significant differences at *P*<0.05. Control: wheat seedlings grown in 7.5 mM NO_3_^−^; SA: wheat seedlings grown in 7.5 mM NH_4_^+^; AN: wheat seedlings grown in 7.5 mM NH_4_^+^ + 1.0 mM NO_3_^−^.

### The root PM H^+^-ATPase activity and the fluxes of H^+^ in the root apex

**Figure 4.**
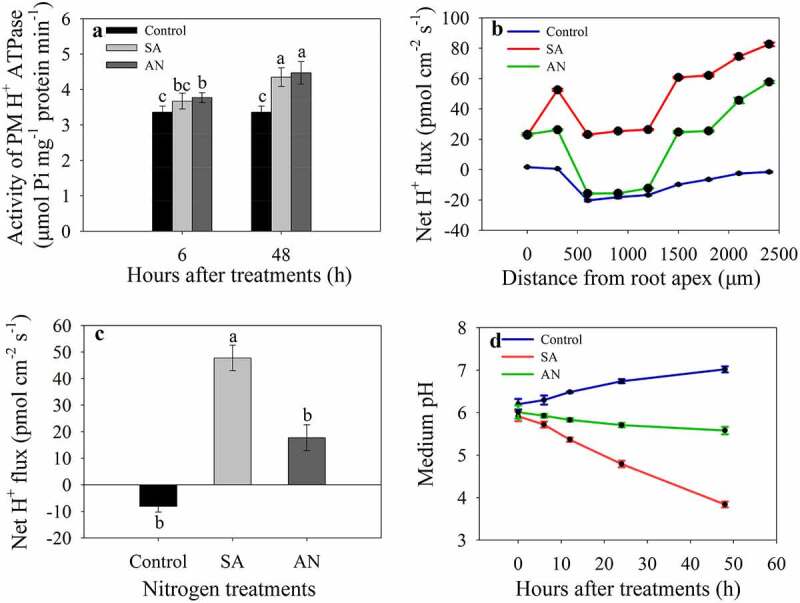
The effects of different N treatments on PM H^+^-ATPase activity (a), the H^+^ flux profile along the root apex (b), the average net H^+^ fluxes along the root apex (c) and the pH value of the hydroponic growth medium (d). b, c: Positive values of the H^+^ flux rate indicate efflux, and negative values indicate influx. The data represent the means of six biological replicates. Different lowercase letters above the columns indicate significant differences at *P* < .05. Control: wheat seedlings grown in 7.5 mM NO_3_^−^; SA: wheat seedlings grown in 7.5 mM NH_4_^+^; AN: wheat seedlings grown in 7.5 mM NH_4_^+^ + 1.0 mM NO_3−._

At both 6 and 48 h, PM H^+^-ATPase activity increased under the SA and AN treatments compared with the control and showed no significant difference between the SA and AN treatments. Under the control conditions, the PM H^+^-ATPase activity remained relatively stable during the period of 6 to 48 h. However, under both the SA and AN conditions, the PM H^+^-ATPase activity was significantly higher at 48 h than at 6 h (Figure 4a).

The rate of H^+^ flux at 48 h was measured along root axes, 0–2,400 μm from the apex, at an interval of 300 μm (Figure 4b), and the average net H^+^ flux along this section of root was calculated for each treatment (Figure 4c). In control seedlings, roots showed net H^+^ influx along the section 0–2,400 μm from the apex. In the SA treatment, the H^+^ showed a strong efflux. After AN treatment, the rate of H^+^ efflux decreased, and moreover, H^+^ showed influx at 500–1,300 μm from the root apex (Figure 4b). As a consequence, the average H^+^ efflux rate 0–2,400 μm from the root apex in the AN treatment was significantly decreased compared with that in the SA treatment (Figure 4c).

### The medium pH

The pH value of the hydroponic growth medium gradually increased from 6.20 to 7.02 under the control conditions during a 48-h culture. However, in the treatment with SA, the medium pH decreased from 5.99 to 3.84 with increasing treatment time. The addition of NO_3_^−^ significantly alleviated the decreases in medium pH compared with the SA treatment, and the medium pH values decreased from 5.95 to 5.58 after 48 h of treatment with AN (Figure 4d).

### Activities of mitochondrial respiratory complexes

**Figure 5.**
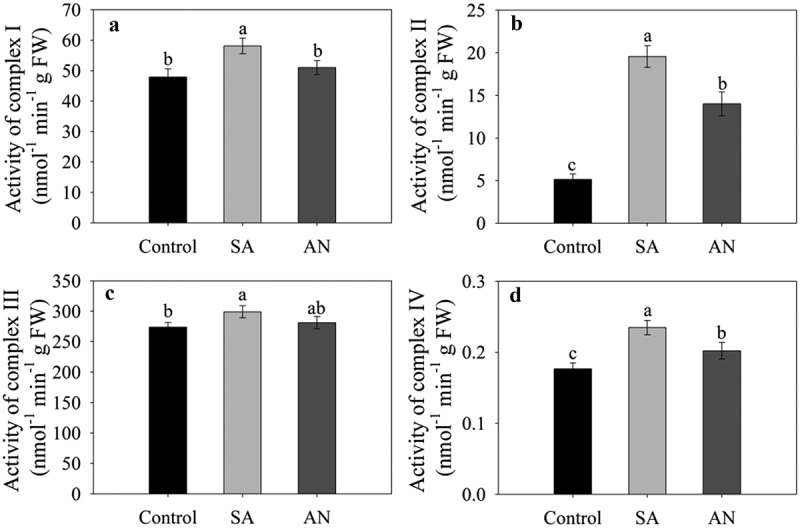
Effects of different N treatments on the activities of root complexes I (a), II (b), III (c), and IV (d) in wheat seedlings. The results represent the mean ± SD of three independent experiments. Different lowercase letters above the columns indicate significant differences at *P* < .05 between experiments. Control: wheat seedlings grown in 7.5 mM NO_3_^−^; SA: wheat seedlings grown in 7.5 mM NH_4_^+^; AN: wheat seedlings grown in 7.5 mM NH_4_^+^ + 1.0 mM NO_3_^−^.

As shown in Figure 5, the activities of root complexes I–IV significantly increased in wheat seedlings in the SA treatment compared with the control. However, the AN treatment significantly decreased the activity of root complexes I, II and IV compared with the NH_4_^+^ only treatment. As a consequence, the activities of complex I and complex III in wheat roots in the AN treatment were similar to those in the control, and the activities of complex II and complex IV in the AN treatment were higher than those in the control.

### Protein carbonylation and lipid peroxidation in roots

**Figure 6.**
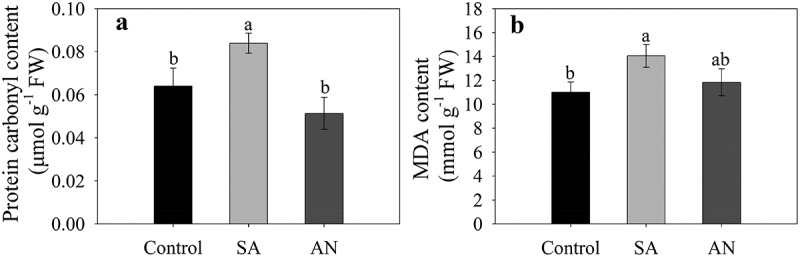
Effects of different N treatments on the protein-bound carbonyl (a) and MDA (b) contents in roots. The results represent the mean ± SD of three independent experiments. Different lowercase letters above the columns indicate significant differences at *P* < .05. Control: wheat seedlings grown in 7.5 mM NO_3_^−^; SA: wheat seedlings grown in 7.5 mM NH_4_^+^; AN: wheat seedlings grown in 7.5 mM NH_4_^+^ + 1.0 mM NO_3_^−^.

The SA treatment significantly increased the content of protein-bound carbonyls and MDA compared to the control. The AN treatment decreased the content of protein-bound carbonyls and MDA to similar levels as in the control (Figure 6).

### Ultrastructure of root meristematic zone cells

**Figure 7.**
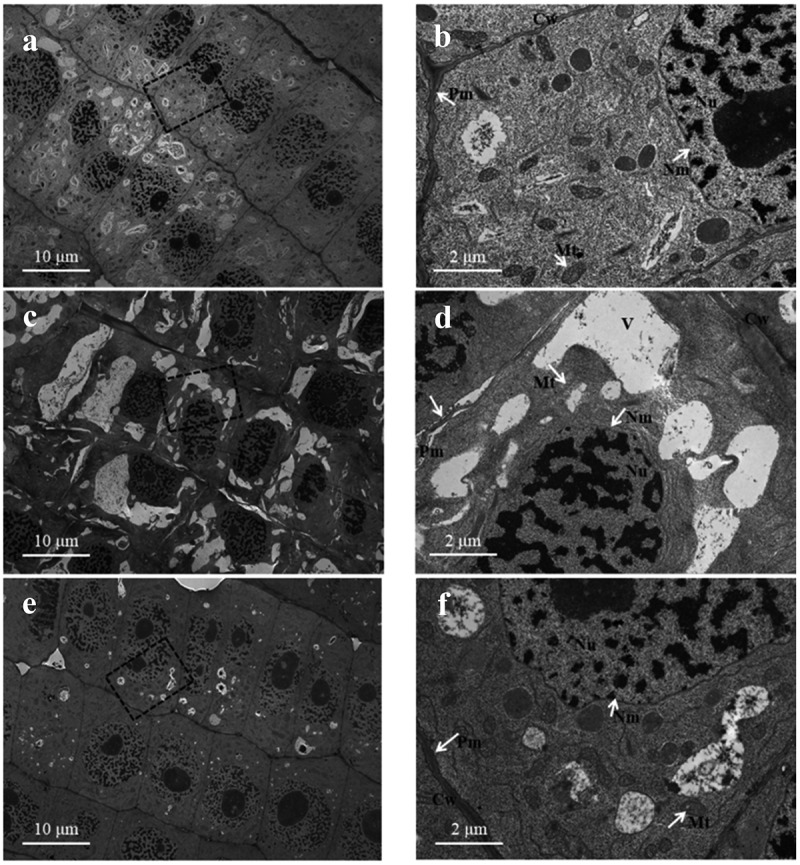
Transmission electron microscopy images of root meristematic cells showing alterations in the cellular structure. a, b: 7.5 mM NO_3_^–^treated wheat seedling roots (control). c, d: 7.5 mM NH_4_^+^-treated roots (SA). e, f: 7.5 mM NH_4_^+^ + 1.0 mM NO_3_^–^treated roots (AN). b, d, and f are higher magnifications of the areas highlighted in a, c, and e, respectively. Cw, cell wall; Mt, mitochondria; Nu, nucleus; Nm, nuclear membrane; Pm, plasma membrane; V, vacuole. Scale bars: 10 µM in a, c, and e; 2 µM in b, d and f.

It is clear that plants grown under control conditions showed typical features, with a complete external envelope and clear boundary of cells belonging to the root meristematic zone. The structures of the nucleolus and nuclear membranes and mitochondria were clearly seen in the cells (Figure 7a, b). Conversely, the ultrastructure of meristem zone cells was obviously changed under SA stress. The cells were irregular, and the integrity of the plasma membranes and nuclear membrane was destroyed. Plasmolysis frequently occurred in the cells, and the cells were highly vacuolated, disorganized and contained a number of indistinct mitochondria compared with control cells (Figure 7c, d). Under the AN treatment, the external envelope and boundary, nucleolus and nuclear membrane were better preserved than under the SA treatment. The mitochondria were more distinct, and the vacuolated cells were significantly reduced compared to the SA treatment (Figure 7e, f).

### Cell viability in the primary root apex

**Figure 8.**
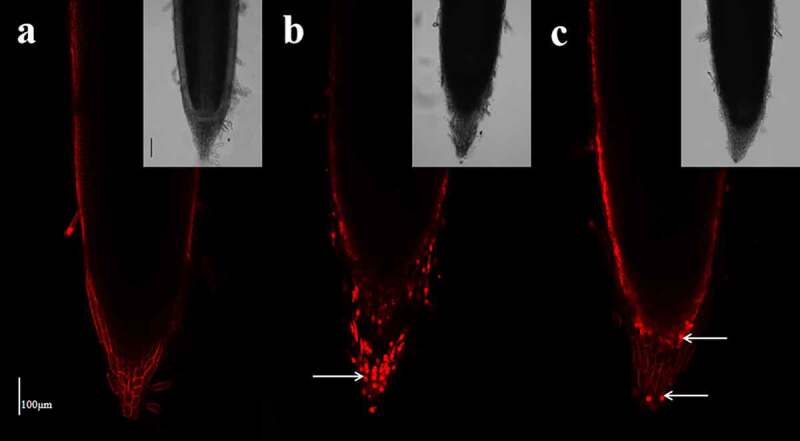
Effect of the different N treatments on the fluorescence of PI in the root tips of wheat seedlings treated with 7.5 mM NO_3_^−^ (a), 7.5 mM NH_4_^+^ (b) and 7.5 mM NH_4_^+^ + 1.0 mM NO_3_^−^ (c). The median view (1,000 μm of the root tip surface) was imaged with the following device settings: wavelength, 1,097 nm; image resolution, 1024 × 1024. The images are representative of the six samples analyzed. The white arrows indicate dead cells. Scale bar = 100 μm.

Fluorescence was visible in only the cell wall of the epidermis and root cap cells in the control roots (Figure 8a). When subjected to SA treatment for 48 h, a large number of root cap cells were stained red with PI (Figure 8b), indicating cell death and cellular toxicity of the high concentration of NH_4_^+^. Under the AN treatment, fluorescence was only detected in a few root cap cells and the wall of the epidermis (Figure 8c).

## Discussion

Ammonium and nitrate are the two forms of inorganic N suitable for plant growth, and excessive NH_4_^+^ concentrations have a highly toxic effect on root growth. The main phenotype of NH_4_^+^ toxicity is characterized by seriously inhibited root growth.^9^ In the present study, we found that treatment with NH_4_^+^ alone significantly decreased the root growth of wheat seedlings and that this inhibition was alleviated by NO_3_^−^ (Figure 1). These results suggest that NO_3_^−^ may alleviate NH_4_^+^ toxicity in the roots of wheat seedlings. This was consistent with the finding that NO_3_^−^ restored the shoot growth in *Arabidopsis thaliana* when exposed to sole NH_4_^+^ treatment.^19^ However, in wheat subjected to sole NH_4_^+^ nutrition, shoot growth was not affected.^21^

There is evidence that NH_4_^+^ toxicity is often accompanied by an accumulation of NH_4_^+^ in plants.^7,19^ Inhibition of NH_4_^+^ uptake and accumulation by roots can alleviate NH_4_^+^ toxicity.^7,33^ In the present study, we examined the NH_4_^+^ content in roots. The results showed that NO_3_^−^ addition did not decrease but increase the root NH_4_^+^ content compared with that in the treatment with only NH_4_^+^ (Figure 2a). Therefore, it is reasonable to assume that the NO_3_^–^dependent alleviation of NH_4_^+^ toxicity may not be through inhibition of NH_4_^+^ accumulation in roots. This view is consistent with the findings that NO_3_^−^ alleviated toxicity symptoms without affecting NH_4_^+^ accumulation in shoots.^19,34^ A most recent study also shows that excessive NH_4_^+^ assimilation by plastidic GLUTAMINE SYNTHETASE 2 rather than NH_4_^+^ accumulation is the primary cause for NH_4_^+^ toxicity.^35^

During N assimilation in plants, the NH_4_^+^ is incorporated into amino acids with the action of NH_4_^+^ assimilation enzymes including glutamine synthetase (GS), glutamate synthase (GOGAT) and glutamate dehydrogenase (GDH), and then converted into protein.^9,18,36^ In a previous work, we found that NO_3_^−^ addition increased the activity of GS, GOGAT and GDH under NH_4_^+^ toxicity.^21^ Therefore, we measured protein content of wheat roots under different N treatments. The results suggested that the roots of wheat seedlings treated with the dual N sources exhibited higher protein content than that treated with sole NH_4_^+^ (Figure 2b). Considering that increased protein content due to NO_3_^−^ addition and that NO_3_^−^ regulates the levels of the enzymes involved in the N assimilation,^37^ we may assume that NO_3_^−^ could alleviate NH_4_^+^ toxicity by promoting the NH_4_^+^ assimilation.

Under high concentrations of NH_4_^+^, an adequate number of carbon skeletons is required for NH_4_^+^ assimilation and amino acid synthesis to prevent overaccumulation and the toxic effects of NH_4_^+^, and carbon skeletons are provided by the TCA cycle.^9,36,38,39^ Thus, the mitochondrial TCA cycle is particularly important for amino acid synthesis and N assimilation.^40^ In the present study, we observed that the activity of CS, aconitase, *α*-KGDH, MDH and PDH and the concentrations of OAA, CA, aconitate, succinic acid, fumaric acid and acetyl-CoA decreased following NH_4_^+^ addition (Figure 3). This result is highly consistent with the findings by Wang et al. (2020),^2^ who reported that NH_4_^+^ alone reduced the levels of TCA cycle intermediates in both shoots and roots and may interrupt the TCA cycle. In the dual N treatment, the activity of these enzymes and the concentration of TCA cycle intermediates were increased compared with those in the treatment with NH_4_^+^ only (Figure 3). Although NO_3_^−^ can not completely eliminate an imbalance between amino acid and organic acid metabolism,^19^ it may partly activate the TCA cycle, increase production of carbon skeletons and thereby alleviate NH_4_^+^ toxicity in wheat seedlings.

PDH catalyses the reactions that convert pyruvate to acetyl-CoA and plays a pivotal role in controlling the entry of carbon into the TCA cycle, a process that does not consume ATP.^41^ The carboxylation of pyruvate to oxaloacetate via PC also replenishes TCA cycle intermediates; however, this process consumes ATP.^42^ In the present study, NH_4_^+^ only treatment decreased the PDH activity but increased the PC activity compared with the NO_3_^−^ only treatment (Figure 3). It has been also reported that the input of pyruvate into the TCA cycle was limited by inactivation of PDH under high NH_4_^+^ nutrition.^19^ Therefore, it is reasonable to speculate that NO_3_^−^ addition may enhance the entry of carbon into the TCA cycle with decreased energy consumption in the root cells, thus contributing to the alleviation of NH_4_^+^ toxicity.

It has been reported that compared to NO_3_^−^ nutrition, NH_4_^+^ nutrition can increase PM H^+^-ATPase activity and cause ionic imbalances and intracellular pH disturbances.^25,43,44^ Our results corroborated this conclusion, as the roots of wheat seedlings treated with NH_4_^+^ exhibited higher PM H^+^-ATPase activity (Figure 4a). PM H^+^-ATPase is responsible for maintaining membrane potential and cytosolic pH homeostasis by pumping protons to the apoplast because during NH_4_^+^ assimilation, a large number of protons are generated in the cytoplasm.^25,33^ Therefore, we measured H^+^ flux in the apical region of roots using NMT. The results revealed that H^+^ showed a strong efflux in the treatment with only NH_4_^+^ (Figure 4b, c), resulting in significant decreases in the medium pH compared to that in the treatment with only NO_3_^−^ (Figure 4d). Previous researches have also showed that when applied as the dominant N source, NH_4_^+^nutrition acidifies the rhizospheric agar medium in rice,^45^ and causes apoplast acidification.^38^ Decreasing external pH is considered as a main response of Arabidopsis plants to high NH_4_^+^ stress.^46^ Furthermore, excessive NH_4_^+^-assimilation caused acidic stress is a primary cause of NH_4_^+^ toxicity.^35^ Based on these data, we postulate that the increased activity of PM H^+^-ATPase (Figure 4a) observed in the treatment with NH_4_^+^ only may contribute to the higher rate of H^+^ efflux (Figure 4b, c) along the root apex and hence medium acidification (Figure 4d), which may be associated with root cytosolic alkalization and thereby, the root growth inhibition.

NO_3_^−^ uptake may alleviate apoplastic acidification caused by NH_4_^+^ toxicity. However, the question of how NO_3_^−^ reduced the rate of net H^+^ efflux and medium acidification remains to be elucidated.^19^ In our study, NO_3_^−^ addition did not affect the activity of PM H^+^-ATPase compared with the addition of NH_4_^+^ alone (Figure 4a), but significantly decreased the rate of net H^+^ efflux and alleviated medium acidification (Figure 4b, c, d). Nitrate transporters (NRTs) in the membrane are primarily responsible for the absorption of NO_3_^−^ by the root system and NO_3_^−^ translocation among different parts of plants.^47–49^ Earlier studies have suggested that most NRTs are NO_3_^−^/H^+^ symporters that can cotransport NO_3_^−^ and H^+^ into cells.^50–53^ Recently, two known NRTs (NRT1s and NRT2s) were shown to function in an H^+^-dependent manner, as they cotransport NO_3_^−^ and H^+^ in the ctenidium of clams (*Tridacna squamosa*).^54^ It is well known that NO_3_^−^ addition to growing media can induce the expression of NO_3_^−^ transporters in plant cells.^55,56^ We therefore propose that NO_3_^−^ addition may induce the symport of H^+^ and NO_3_^−^ into root cells, decrease net H^+^ efflux, reduce cytosolic alkalization and rhizospheric environment acidification and thus alleviate NH_4_^+^ toxicity. This review is supported by the findings that NO_3_^−^ transporter NRT1.1-mediated NO_3_^−^ uptake contributes to low-pH stress tolerance in plant,^57^ and NRTs and SLAH3 may maintain sufficient levels of NO_3_^−^ in the rhizosphere to buffer the low pH caused by NH_4_^+^ stress.^6^

High NH_4_^+^ application increased respiratory activity in NH_4_^+^-supplied plants and caused higher mitochondrial reactive oxygen species (ROS) formation, leading to oxidative stress.^58,59^ The production of O_2_˙^−^ and H_2_O_2_ is mainly caused by the mitochondrial respiratory chain under stress, and the maximum O_2_˙^−^ production rate *in vivo* is proportional to the content of respiratory complexes.^60,61^ In particular, complexes I, II, and III of the mitochondrial respiratory chain have been identified as production sites of O_2_˙^−^.^60,62^ A previous study indicates that nitrate could decrease in ROS accumulation by reducing ROS production.^63^ In our previous study, we found that the addition of only NH_4_^+^ appeared to increase H_2_O_2_ and O_2_˙^−^ contents compared to the addition of the same concentration of NO_3_^−^ and that addition of NO_3_^−^ decreased H_2_O_2_ and O_2_˙^−^ contents compared to the sole NH_4_^+^ treatment.^21^ However, how the mechanisms by which high NH_4_^+^ addition increases ROS production and whether NO_3_^−^ alleviates NH_4_^+^ toxicity by reducing oxidative stress is unclear.^19^ Previous studies that the activities of succinate dehydrogenase, cytochrome *c* oxidoreductase and cytochrome *c* oxidase were increased more or less during NH_4_^+^ toxicity.^64,65^ In the present study, we assayed the activities complex I–IV, and found that the addition of only NH_4_^+^ enhanced complex I–IV activities compared to the control, and NO_3_^−^ addition decreased the activities of complexes I–IV in wheat roots compared with the addition of NH_4_^+^ only; in particular, the activities of complex I and complex III were similar to those in the control (Figure 5). Protein carbonylation on amino acid side chains and lipid peroxidation (measured as the decomposition product MDA) have been regarded as important indicators of oxidative damage.^31,66,67^ In the present study, the NH_4_^+^ only supply enhanced the contents of protein-bound carbonyls and MDA, while NO_3_^−^ addition decreased the contents of protein-bound carbonyls and MDA to levels similar to those in the control (Figure 6). These results are highly consistent with previous reports on *Arabidopsis* and submerged macrophytes, where excessive NH_4_^+^ can cause an increase in protein-bound carbonyl and MDA contents.^4,68^ Similarly, it has been also reported that NO_3_^–^-fed roots produced lower levels of ROS than NH_4_^+^-fed roots.^69^ Based on these data, we speculate that NO_3_^−^ addition decreased the activities of mitochondrial respiratory chain complexes and the ROS production, thus alleviating oxidative damage to proteins and lipids.

Oxidative damage induces DNA damage, lipid peroxidation, protein modification, and organelle ultrastructure changes and affects cell viability.^70,71^ In the present study, we observed that sole NH_4_^+^ caused vacuolation and membrane disruption in cells of the root meristem zone (Figure 7c, d). NO_3_^−^ addition ameliorated the membrane integrity and reduced the ultrastructure disruption of root cells (Figure 7e, f). In addition, we tested the effect of different N sources on root cell viability By staining with PI, which is a vital staining reagent that stains cell walls and nucleic acids and cannot penetrate the plasma membranes of living cells but can traverse the broken membrane of dead cells.^72^ We found that compared with high viability of root cap cells of the control wheat seedlings (Figure 8a), a number of dead root cap cells were detected in the plants exposed to NH_4_^+^ alone, as indicated by strong fluorescence (Figure 8b). Additional NO_3_^−^ increased cell viability compared to that in the treatment with only NH_4_^+^ (Figure 8c). The results presented here suggest that additional NO_3_^−^ may significantly improve root cell viability likely by relieving sole NH_4_^+^ induced oxidative damage to root cells.

## Conclusions

Compared with sole NH_4_^+^ nutrition, NO_3_^−^ addition increases in enzyme activity and the concentration of most TCA cycle intermediates and thereby promotes the production of carbon skeletons for N assimilation. Moreover, NO_3_^−^ addition reduces the rate of net H^+^ efflux and alleviates solution acidification compared with the NH_4_^+^ only treatment, which may improve the ability of root cells to maintain cytosolic pH homeostasis. Furthermore, the addition of NO_3_^−^ decreases activity of mitochondrial respiratory complexes, mitigates cell oxidative damage and increases cell viability. Ultimately, NO_3_^−^ addition boosts root growth under high concentrations of NH_4_^+^ in wheat seedlings.

## Supplementary Material

Supplemental Material

## References

[1] Escobar MA, Geisler DA, Rasmusson AG. Reorganization of the alternative pathways of the *Arabidopsis* respiratory chain by nitrogen supply: opposing effects of ammonium and nitrate. Plant J. 2006;45(5):1–13. doi:10.1111/j.1365-313X.2005.02640.x. 16460511

[2] Wang F, Gao J, Yong JW, Liu Y, Cao D, He X. Glutamate over-accumulation may serve as an endogenous indicator of tricarboxylic acid (TCA) cycle suppression under NH_4_^+^ nutrition in wheat (*Triticum aestivum* L.) seedlings. Environ Exp Bot. 2020;177:104–130. doi:10.1016/j.envexpbot.2020.104130.

[3] Hernández-Pérez A, Valdez-Aguilar LA, Villegas-Torres OG, Alía-Tejacal I, Trejo-Téllez LI, Sainz-Aispuro MDJ. Effects of ammonium and calcium on lisianthus growth. Hortic Environ Biote. 2016;57(2):123–131. doi:10.1007/s13580-016-0004-1.

[4] Gao J, Ren P, Zhou Q, Zhang J. Comparative studies of the response of sensitive and tolerant submerged macrophytes to high ammonium concentration stress. Aquat Toxicol. 2019;211:57–65. doi:10.1016/j.aquatox.2019.03.020. 30952066

[5] Ou X, Cui X, Zhu D, Guo L, Liu D, Yang Y. Cultivation mode of *Panax notoginseng* causes NH_4_^+^ accumulation in planting soil. Arch Agron Soil Sci. 2021;67:960–973. doi:10.1080/03650340.2020.1771314.

[6] Zheng X, He K, Kleist T, Chen F, Luan S. Anion channel SLAH 3 functions in nitrate dependent alleviation of ammonium toxicity in *Arabidopsis*. Plant Cell Environ. 2015;38:474–486. doi:10.1111/pce.12389. 24944085

[7] Ma X, Zhu C, Yang N, Gan L, Xia K. *γ*-Aminobutyric acid addition alleviates ammonium toxicity by limiting ammonium accumulation in rice (*Oryza sativa*) seedlings. Physiol Plantarum. 2016;158:389–401. doi:10.1111/ppl.12473. 27218863

[8] Ariz I, Esteban R, García-Plazaola JI, Becerril JM, Aparicio-Tejo PM, Moran JF. High irradiance induces photoprotective mechanisms and a positive effect on NH_4_^+^ stress in *Pisum sativum* L. J Plant Physiol. 2010;167:1038–1045. doi:10.1016/j.jplph.2010.02.014. 20434233

[9] Britto DT, Kronzucker HJ. NH_4_^+^ toxicity in higher plants: a critical review. J Plant Physiol. 2002;159:567–584.

[10] Roosta HR, Schjoerring JK. Effects of ammonium toxicity on nitrogen metabolism and elemental profile of cucumber plants. J Plant Nutr. 2007;30(11):1933–1951. doi:10.1080/01904160701629211.

[11] Da Silva GP, de Mello Prado R, Ferreira RPS. Absorption of nutrients, growth and nutritional disorders resulting from ammonium toxicity in rice and spinach plants. Emi J Food Agr. 2016;28(12):882–889. doi:10.9755/ejfa.2016-09-1294.

[12] Viciedo DO, de Mello Prado R, Toledo RL, dos Santos LCN, Hurtado AC, Nedd LLT, Gonzalez LC. Silicon supplementation alleviates ammonium toxicity in sugar beet (*Beta vulgaris* L.). J Soil Sci Plant Nut. 2019;19:413–419. doi:10.1007/s42729-019-00043-w.

[13] Sun D, Fang X, Xiao C, Ma Z, Huang X, Su J, Li J, Wang J, Wang S, Luan S, et al. Kinase SnRK1.1 regulates nitrate channel SLAH3 engaged in nitrate-dependent alleviation of ammonium toxicity. Plant Physiol. 2021;186(1):731–749. doi:10.1093/plphys/kiab057. 33560419 PMC8154061

[14] Barreto RF, Júnior AAS, Maggio MA. de Mello Prado R. Silicon alleviates ammonium toxicity in cauliflower and in broccoli. Sci Hortic-Amsterdam. 2017;225:743–750. doi:10.1016/j.scienta.2017.08.014.

[15] Vega-Mas I, Pérez-Delgado CM, Marino D, Fuertes-Mendizábal T, González-Murua C, Márquez AJ, Betti M, Estavillo JM, González-Moro MB. Elevated CO_2_ induces root defensive mechanisms in tomato plants when dealing with ammonium toxicity. Plant Cell Physiol. 2017;58:2112–2125. doi:10.1093/pcp/pcx146. 29059445

[16] Magalhaes JR, Huber DM, Tsai CY. Evidence of increased ^15^N-ammonium assimilation in tomato plants with exogenous *α*-ketoglutarate. Plant Sci. 1992;85(2):135–141. doi:10.1016/0168-9452(92)90108-X.

[17] Roosta HR, Schjoerring JK. Effects of nitrate and potassium on ammonium toxicity in cucumber plants. J Plant Nutr. 2008;31(7):1270–1283. doi:10.1080/01904160802135050.

[18] Ou X, Li S, Liao P, Cui X, Zheng B, Yang Y, Liu D, Zheng Y. The transcriptome variations of *Panax notoginseng* roots treated with different forms of nitrogen fertilizers. BMC Genomics. 2019;20(S9):1–15. doi:10.1186/s12864-019-6340-7. 31874632 PMC6929466

[19] Hachiya T, Watanabe CK, Fujimoto M, Ishikawa T, Takahara K, Kawai-Yamada M, Uchimiya H, Uesono Y, Terashima I, Noguchi K. Nitrate addition alleviates ammonium toxicity without lessening ammonium accumulation, organic acid depletion and inorganic cation depletion in *Arabidopsis thaliana* shoots. Plant Cell Physiol. 2012;53(3):577–591. doi:10.1093/pcp/pcs012. 22318863

[20] Sasaki K, Kojima S. Identification of genomic regions regulating ammonium-dependent inhibition of primary root length in *Arabidopsis thaliana*. Soil Sci Plant Nutr. 2018;64(6):746–751. doi:10.1080/00380768.2018.1524268.

[21] Zhang Y, Zhang YX, Lü XM, Du WY, Feng B, Li HW, Wang ZS, Xia HY, Fan SJ, Kong LA. Study on physiological mechanism of NO_3_^−^ alleviating NH_4_^+^ stress in wheat. Plant Physiol J. 2021;57:480–492.

[22] Hoagland DR, Arnon DI. The water-culture method for growing plants without soil. Calif Agric Exp St, Circ. 1950;347:1–32. 2nd edit.

[23] Balkos KD, Britto DT, Kronzucker HJ. Optimization of ammonium acquisition and metabolism by potassium in rice (*Oryza sativa* L. Cv. IR‐72). Plant Cell Environ 2010;33:23–34. 19781010 10.1111/j.1365-3040.2009.02046.x

[24] Bradford MM. A rapid and sensitive method for the quantitation of microgram quantities of protein utilizing the principle of protein-dye binding. Anal Biochem. 1976;72(1–2):248–254. doi:10.1016/0003-2697(76)90527-3. 942051

[25] Zhang M, Ding M, Xu F, Afzal MR, Chen X, Zeng H, Yan F, Zhu Y. Involvement of plasma membrane H^+^-ATPase in the ammonium-nutrition response of barley roots. J Plant Nutr Soil Sc. 2018;181:878–885. doi:10.1002/jpln.201800045.

[26] Baginski ES, Foa PP, Zak B. Determination of phosphate: study of labile organic phosphate interference. Clin Chim Acta. 1967;15(1):155–158. doi:10.1016/0009-8981(67)90340-3.

[27] Eubel H, Heazlewood JL, Millar AH. Isolation and subfractionation of plant mitochondria for proteomic analysis. Plant Proteomics. Humana Press, America. 2007;335:49–62. 10.1385/1-59745-227-0:49 17093302

[28] Verner Z, Čermáková P, Škodová I, Kováčová B, Lukeš J, Horváth A. Comparative analysis of respiratory chain and oxidative phosphorylation in *Leishmania tarentolae, Crithidia fasciculata, Phytomonas serpens* and procyclic stage of *Trypanosoma brucei*. Mol Biochem Parasit. 2014;193(1):55–65. doi:10.1016/j.molbiopara.2014.02.003. 24556248

[29] Sudheesh NP, Ajith TA, Janardhanan KK. *Ganoderma lucidum* (Fr.) P. Karst enhances activities of heart mitochondrial enzymes and respiratory chain complexes in the aged rat. Biogerontology. 2009;10(5):627–636. doi:10.1007/s10522-008-9208-9. 19123066

[30] Singh N, Hroudová J, Fišar Z. Cannabinoid-induced changes in the activity of electron transport chain complexes of brain mitochondria. J Mol Neurosci. 2015;56(4):926–931. doi:10.1007/s12031-015-0545-2. 25820672

[31] Hachiya T, Watanabe CK, Boom C, Tholen D, Takahara K, Kawai-Yamada M, Uchimiya H, Uesono Y, Terashima I, Noguchi K. Ammonium-dependent respiratory increase is dependent on the cytochrome pathway in *Arabidopsis thaliana* shoots. Plant Cell Environ. 2010;33(11):1888–1897. doi:10.1111/j.1365-3040.2010.02189.x. 20545883

[32] Bouabid K, Lamchouri F, Toufik H, Faouzi MEA. Phytochemical investigation, in vitro and in vivo antioxidant properties of aqueous and organic extracts of toxic plant: *atractylis gummifera* L. J Ethnopharmacol. 2020;253:112640. doi:10.1016/j.jep.2020.112640. 32027998

[33] Weng L, Zhang M, Wang K, Chen G, Ding M, Yuan W, Zhu Y, Xu W, Xu F. Potassium alleviates ammonium toxicity in rice by reducing its uptake through activation of plasma membrane H^+^-ATPase to enhance proton extrusion. Plant Physiol Bioch. 2020;151:429–437. doi:10.1016/j.plaphy.2020.03.040. 32289636

[34] Yang H, von der Fecht-Bartenbach J, Friml J, Lohmann JU, Neuhäuser B, Ludewig U. Auxin-modulated root growth inhibition in *Arabidopsis thaliana* seedlings with ammonium as the sole nitrogen source. Funct Plant Biol. 2015;42(3):239–251. doi:10.1071/FP14171. 32480670

[35] Hachiya T, Inaba J, Wakazaki M, Sato M, Toyooka K, Miyagi A, Kawai-Yamada M, Sugiura D, Nakagawa T, Kiba T, et al. Excessive ammonium assimilation by plastidic glutamine synthetase causes ammonium toxicity in *Arabidopsis thaliana*. Nat Commun. 2021;12(1):4944. doi:10.1038/s41467-021-25238-7. 34400629 PMC8367978

[36] Liu X, Wang K, Zhang J, Wang J, Wu J, Peng F. Ammonium removal potential and its conversion pathways by free and immobilized *Scenedesmus obliquus* from wastewater. Bioresource Technol. 2019;283:184–190. doi:10.1016/j.biortech.2019.03.038. 30904698

[37] Yoneyama T, Suzuki A. Exploration of nitrate-to-glutamate assimilation in non-photosynthetic roots of higher plants by studies of ^15^N-tracing, enzymes involved, reductant supply, and nitrate signaling: a review and synthesis. Plant Physiol Bioch. 2019;136:245–254. doi:10.1016/j.plaphy.2018.12.011. 30710774

[38] Feng H, Fan X, Miller AJ, Xu G. Plant nitrogen uptake and assimilation: regulation of cellular pH homeostasis. J Exp Bot. 2020;71:4380–4392. doi:10.1093/jxb/eraa150. 32206788 PMC7382382

[39] Setién I, Fuertes-Mendizabal T, González A, Aparicio-Tejo PM, González-Murua C, González-Moro MB, Estavillo JM. High irradiance improves ammonium tolerance in wheat plants by increasing N assimilation. J Plant Physiol. 2013;170(8):758–771. doi:10.1016/j.jplph.2012.12.015. 23485260

[40] Szal B, Podgórska A. The role of mitochondria in leaf nitrogen metabolism. Plant Cell Environ. 2012;35(10):1756–1768. doi:10.1111/j.1365-3040.2012.02559.x. 22697909

[41] Yu H, Du X, Zhang F, Zhang F, Hu Y, Liu S, Jiang X, Wang G, Liu D. A mutation in the E2 subunit of the mitochondrial pyruvate dehydrogenase complex in *Arabidopsis* reduces plant organ size and enhances the accumulation of amino acids and intermediate products of the TCA cycle. Planta. 2012;236(2):387–399. doi:10.1007/s00425-012-1620-3. 22391856

[42] Strickaert A, Corbet C, Spinette SA, Craciun L, Dom G, Andry G, Larsimont D, Wattiez R, Dumont JE, Feron O, et al. Reprogramming of energy metabolism: increased expression and roles of pyruvate carboxylase in papillary thyroid cancer. Thyroid. 2019;29(6):845–857. doi:10.1089/thy.2018.0435. 30990120

[43] Zeng H, Liu G, Kinoshita T, Zhang R, Zhu Y, Shen Q, Xu G. Stimulation of phosphorus uptake by ammonium nutrition involves plasma membrane H^+^-ATPase in rice roots. Plant Soil. 2012;357:205–214.

[44] Carr NF, Boaretto RM, Mattos J. Coffee seedlings growth under varied NO_3_^−^: NH_4_^+^ ratio: consequences for nitrogen metabolism, amino acids profile, and regulation of plasma membrane H^+^-ATPase. Plant Physiol Bioch. 2020;154:11–20. doi:10.1016/j.plaphy.2020.04.042. 32516683

[45] Geilfus CM. The pH of the apoplast: dynamic factor with functional impact under stress. Mol Plant. 2017;10(11):1371–1386. doi:10.1016/j.molp.2017.09.018. 28987886

[46] Patterson K, Cakmak T, Cooper A, Lager IDA, Rasmusson AG, Escobar MA. Distinct signalling pathways and transcriptome response signatures differentiate ammonium-and nitrate-supplied plants. Plant Cell Environ. 2010;33:1486–1501. 20444219 10.1111/j.1365-3040.2010.02158.x PMC2920365

[47] Léran S, Muños S, Brachet C, Tillard P, Gojon A, Lacombe B. *Arabidopsis* NRT1. 1 is a bidirectional transporter involved in root-to-shoot nitrate translocation. Mol Plant. 2013;6:1984–1987. doi:10.1093/mp/sst068. 23645597

[48] Zhang GB, Meng S, Gong JM. The expected and unexpected roles of nitrate transporters in plant abiotic stress resistance and their regulation. Int J Mol Sci. 2018;19(11):3535–3550. doi:10.3390/ijms19113535. PMC6274899 30423982

[49] Li M, Tian H, Gao Y. A genome-wide analysis of NPF and NRT2 transporter gene families in bread wheat provides new insights into the distribution, function, regulation and evolution of nitrate transporters. Plant Soil. 2021;465(1–2):47–63. doi:10.1007/s11104-021-04927-8.

[50] Britto DT, Kronzucker HJ. Nitrogen acquisition, PEP carboxylase, and cellular pH homeostasis: new views on old paradigms. Plant Cell Environ. 2005;28(11):1396–1409. doi:10.1111/j.1365-3040.2005.01372.x.

[51] Wang YY, Hsu PK, Tsay YF. Uptake, allocation and signaling of nitrate. Trends Plant Sci. 2012;17(8):458–467. doi:10.1016/j.tplants.2012.04.006. 22658680

[52] Xu G, Fan X, Miller AJ. Plant nitrogen assimilation and use efficiency. Annu Rev Plant Biol. 2012;63(1):153–182. doi:10.1146/annurev-arplant-042811-105532. 22224450

[53] Luo J, Qin J, He F, Li H, Liu T, Polle A, Peng C, Luo ZB. Net fluxes of ammonium and nitrate in association with H^+^ fluxes in fine roots of *Populus popularis*. Planta. 2013;237(4):919–931. doi:10.1007/s00425-012-1807-7. 23179443

[54] Ip YK, Hiong KC, Teng JH, Boo MV, Choo CY, Wong WP, Chew SF. The fluted giant clam (*Tridacna squamosa*) increases nitrate absorption and upregulates the expression of a homolog of SIALIN (H^+^:2NO_3_^−^ cotransporter) in the ctenidium during light exposure. Coral Reefs. 2020;39:451–465. doi:10.1007/s00338-020-01907-9.

[55] Mantelin S, Touraine B. Plant growth-promoting bacteria and nitrate availability: impacts on root development and nitrate uptake. J Exp Bot. 2004;55:27–34. doi:10.1093/jxb/erh010. 14623902

[56] Hu Y, Fernández V. Nitrate transporters in leaves and their potential roles in foliar uptake of nitrogen dioxide. Front Plant Sci. 2014;5:360–369. doi:10.3389/fpls.2014.00360. 25126090 PMC4115617

[57] Fang XZ, Tian WH, Liu XX, Lin XY, Jin CW, Zheng SJ. Alleviation of proton toxicity by nitrate uptake specifically depends on nitrate transporter 1.1 in *Arabidopsis*. New Phytol. 2016;211(1):149–158. doi:10.1111/nph.13892. 26864608

[58] Guo S, Schinner K, Sattelmacher B, Hansen UP. Different apparent CO_2_ compensation points in nitrate-and ammonium-grown *Phaseolus vulgaris* and the relationship to non-photorespiratory CO_2_ evolution. Physiol Plantarum. 2005;123:288–301. doi:10.1111/j.1399-3054.2005.00467.x.

[59] Podgórska A, Ostaszewska-Bugajska M, Borysiuk K, Tarnowska A, Jakubiak M, Burian M, Rasmusson AG, Szal B. Suppression of external NADPH dehydrogenase-NDB1 in *Arabidopsis thaliana* confers improved tolerance to ammonium toxicity via efficient glutathione/redox metabolism. Int J Mol Sci. 2018;19:1412–1437. doi:10.3390/ijms19051412. PMC5983774 29747392

[60] Murphy MP. How mitochondria produce reactive oxygen species. Biochem J. 2009;417(1):1–13. doi:10.1042/BJ20081386. 19061483 PMC2605959

[61] Jing H, Liu H, Zhang L, Gao J, Song H, Tan X. Ethanol induces autophagy regulated by mitochondrial ROS in *Saccharomyces cerevisiae*. J Microbiol Biotechn. 2018;28(12):1982–1991. doi:10.4014/jmb.1806.06014. 30394045

[62] Xue C, Li X, Liu G, Liu W. Evaluation of mitochondrial respiratory chain on the generation of reactive oxygen species and cytotoxicity in HACAT cells induced by nanosized titanium dioxide under UVA irradiation. Int J Toxicol. 2016;35(6):644–653. doi:10.1177/1091581816661853. 27503939

[63] Zang L, Morère-Le Paven MC, Clochard T, Porcher A, Satour P, Mojović M, Vidović M, Limami AM, Montrichard F. Nitrate inhibits primary root growth by reducing accumulation of reactive oxygen species in the root tip in *Medicago truncatula*. Plant Physiol Bioch. 2020;146:363–373. doi:10.1016/j.plaphy.2019.11.006. 31786508

[64] Matsumoto H, Wakiuchi N, Takahashi E. Changes of some mitochondrial enzyme activities of cucumber leaves during ammonium toxicity. Physiol Plantarum. 1971;25(3):353–357. doi:10.1111/j.1399-3054.1971.tb01454.x.

[65] Matsumoto H, Tamura K. Respiratory stress in cucumber roots treated with ammonium or nitrate nitrogen. Plant Soil. 1981;60(2):195–204. doi:10.1007/BF02374104.

[66] Ciacka K, Tymiński M, Gniazdowska A, Krasuska U. Carbonylation of proteins-an element of plant ageing. Planta. 2020;252(1):1–13. doi:10.1007/s00425-020-03414-1. 32613330 PMC7329788

[67] Qi DU, Zhao XH, Xia L, Jiang CJ, Wang XG, Han Y, Wang J, Yu HQ. Effects of potassium deficiency on photosynthesis, chloroplast ultrastructure, ROS, and antioxidant activities in maize (*Zea mays* L.). J Integr Agr. 2019;18(2):395–406. doi:10.1016/S2095-3119(18)61953-7.

[68] Podgórska A, Gieczewska K, Łukawska-Kuźma K, Rasmusson AG, Gardeström P, Szal B. Long-term ammonium nutrition of *Arabidopsis* increases the extra chloroplastic NAD(P)H/NAD(P)^+^ ratio and mitochondrial reactive oxygen species level in leaves but does not impair photosynthetic capacity. Plant Cell Environ. 2013;36:2034–2045. 23574048 10.1111/pce.12113

[69] Wany A, Gupta AK, Kumari A, Mishra S, Singh N, Pandey S, Vanvari R, Igamberdiev AU, Fernie AR, Gupta KJ. Nitrate nutrition influences multiple factors in order to increase energy efficiency under hypoxia in *Arabidopsis*. Ann Bot-London. 2019;123(4):691–705. doi:10.1093/aob/mcy202. PMC6417481 30535180

[70] Jomova K, Valko M. Advances in metal-induced oxidative stress and human disease. Toxicology. 2011;283(2–3):65–87. doi:10.1016/j.tox.2011.03.001. 21414382

[71] Huang H, Ullah F, Zhou D-X, Yi M, Zhao Y. Mechanisms of ROS regulation of plant development and stress responses. Front Plant Sci. 2019;10:800–810. doi:10.3389/fpls.2019.00800. 31293607 PMC6603150

[72] Perrine-Walker F, Le K. Propidium iodide enabled live imaging of *Pasteuria* sp.-*Pratylenchus zeae* infection studies under fluorescence microscopy. Protoplasma. 2021;258(2):279–287. doi:10.1007/s00709-020-01567-0. 33070241

