# Supplementary material for: Nitrate alleviates ammonium toxicity in wheat (Triticum aestivum L.) by regulating tricarboxylic acid cycle and reducing rhizospheric acidification and oxidative damage
Source: Plant Signal Behav. 2021 Nov 9;16(12):1991687. doi: 10.1080/15592324.2021.1991687 (PMC9208799; doi:10.1080/15592324.2021.1991687)
Supplement: Supplemental Material [file KPSB_A_1991687_SM7451.docx]

**Table S1.** Activities of enzymes and concentrations of intermediates of three treatments

|  | control | SA | AN |
| --- | --- | --- | --- |
| citrate synthase | 16.08±0.46^a^ | 5.40±0.46^c^ | 14.04±0.07^b^ |
| citric acid | 1088.39±18.40^a^ | 626.29±18.47^c^ | 953.69±17.34^b^ |
| malate dehydrogenase | 4120.54±98.01^a^ | 3657.82±106.69^b^ | 4239.72±96.65^a^ |
| oxaloacetic acid | 416.55±1.59^a^ | 392.39±1.71^b^ | 418.69±2.05^a^ |
| fumaric acid | 211.66±2.99^a^ | 106.70±2.95^c^ | 178.52±2.19^b^ |
| α-ketoglutarate dehydrogenase | 18.35±0.99^a^ | 12.40±0.32^b^ | 19.99±0.82^a^ |
| succinic acid | 576.72±5.34^a^ | 344.58±4.43^c^ | 509.43±12.98^b^ |
| aconitase | 27.13±0.73^a^ | 16.71±0.38^c^ | 24.03±0.35^b^ |
| aconitate | 1023.65±36.11^a^ | 738.85±14.45^c^ | 929.50±12.10^b^ |
| succino dehydrogenase | 2.28±0.01^c^ | 3.91±0.18^a^ | 2.78±0.17^b^ |
| acetyl coenzyme A | 20.78±0.92^a^ | 16.58±0.79^b^ | 22.44±1.27^a^ |
| pyruvate dehydrogenase | 3.82±0.15^a^ | 3.08±0.16^b^ | 3.54±0.18^a^ |
| Pyruvate carboxylase | 78.47±1.77^b^ | 90.95±3.06^a^ | 79.48±2.85^b^ |
| malic acid | 388.83±7.89^a^ | 118.41±1.08^c^ | 269.62±3.10^b^ |

Effects of different N treatments on activities of enzymes and concentrations of the contents of intermediates in wheat seedling roots. The results represent the mean ± SD of three independent experiments. Different lowercase letters in each row indicate significant differences at *P*<0.05. Control: wheat seedlings grown in 7.5 mM NO_3_^-^; SA: wheat seedlings grown in 7.5 mM NH_4_^+^; AN: wheat seedlings grown in 7.5 mM NH_4_^+^ + 1.0 mM NO_3_^-^.
